# Data on coding association rules from an inpatient administrative health data coded by International classification of disease - 10th revision (ICD-10) codes

**DOI:** 10.1016/j.dib.2018.02.043

**Published:** 2018-02-16

**Authors:** Mingkai Peng, Vijaya Sundararajan, Tyler Williamson, Evan P. Minty, Tony C. Smith, Chelsea T.A. Doktorchik, Hude Quan

**Affiliations:** a Department of Community Health Sciences, University of Calgary, Calgary, Canada; b Department of Medicine, St. Vincent's Hospital, University of Melbourne, Melbourne, Australia; c Cumming School of Medicine, University of Calgary, Calgary, Canada; d Department of Computer Science, University of Waikato, Hamilton, New Zealand

## Abstract

Data presented in this article relates to the research article entitled “Exploration of association rule mining for coding consistency and completeness assessment in inpatient administrative health data” (Peng et al. [1]) in preparation).

We provided a set of ICD-10 coding association rules in the age group of 55 to 65. The rules were extracted from an inpatient administrative health data at five acute care hospitals in Alberta, Canada, using association rule mining. Thresholds of support and confidence for the association rules mining process were set at 0.19% and 50% respectively. The data set contains 426 rules, in which 86 rules are not nested. Data are provided in the supplementary material. The presented coding association rules provide a reference for future researches on the use of association rule mining for data quality assessment.

**Specifications Table**

**Table:** 

Subject area	Medicine
More specific subject area	International Classification of Disease – 10^th^ revision (ICD-10) diagnosis codes in hospital setting
Type of data	Table
How data was acquired	Administrative health data coded by professional coders at acute care hospitals
Data format	Analyzed
Experimental factors	Association rule mining was conducted on an inpatient administrative health data at the age group of 55 to 65 to extract the coding association rules
Experimental features	Thresholds of support and confidence for association rule mining were set at 0.19% and 50% respectively.
Data source location	Alberta, Canada
Data accessibility	Data submitted with this article

**Value of the data**

• Data could be used to assess data quality in ICD-10 coded health data.
• These data provide the reference for the future studies on the development of data quality rules in observational health data using association rule mining.
• These data will make it possible to improve the quality of studies using ICD-10 coded data for evidence generation, by understanding the associations hidden in the data.
• Data on association rules can be used as a cost-effective way to improve the quality of data collection in hospital settings.

## 1 Data

ICD-10 classification system has been used by many countries for coding cause of death and for hospital morbidities as mandated by World Health Organization (WHO). We provided a set of ICD-10 coding association rules in the age group of 55 to 65 learned from an inpatient administrative health data [1]. In total, there were 426 rules with 86 rules not nested in the other rules. The rules captured meaningful clinical associations hidden in the database.

## 2 Experimental design, materials and methods

We used Alberta hospital discharge abstract data (DAD) for association rule mining. Following the guideline developed by the Canadian Institution of Health Information (CIHI), hospital coders abstract clinical documents (e.g. discharge summary) using ICD-10, Canada (ICD-10-CA) classification system into diagnosis codes. ICD-10-CA was developed by CIHI based on the ICD-10 classification terminology from WHO by adding one or more digits for some diagnosis codes. For each hospital admission, a coder can assign up to 25 ICD-10-CA codes. We extracted 26378 DAD records at the age group of 55 to 65 from 5 acute care hospitals in 2013. The ICD-10-CA diagnosis codes were mapped back to ICD-10 for international generalizability before analysis.

Association rule mining is the process of finding clinical and interesting associations or patterns hidden in data. An association rule is an expression of X → Y, where X and Y are disjoint and nonempty code sets. Code sets of X and Y are the left-hand side (LHS) and right-hand side (RHS) of the rule, respectively. The strength of an association rule can be measured in terms of support and confidence. The Apriori algorithm implemented in R package *arules* was used for association rule mining on ICD-10 codes [2]. The thresholds of support and confidence for association rule mining were set at 0.19% and 50% respectively. Bootstrapping was used in the rule mining process to ensure generalizability of the developed rules. Nested rules are identified, with two rules being considered nested if they have the same RHS and the LHS of one rule is a subset of the LHS of the other rule. For example, two rules of X → Y and {X, Z} → Y are nested. In total, there were 426 rules with 86 rules not nested. The support and confidence of rules were presented in the data. We also included the values of two commonly used measures: lift and conviction [3]. Description of each variable in the data are provided. The data are submitted as supplementary materials in excel format with the article.
